# Supplementary material for: Lysosomal glucocerebrosidase is needed for ciliary Hedgehog signaling: A convergent pathway contributing to Parkinson’s disease
Source: Proc Natl Acad Sci U S A. 2025 Jul 30;122(31):e2504774122. doi: 10.1073/pnas.2504774122 (PMC12337309; doi:10.1073/pnas.2504774122)
Supplement: Supplementary file 1 — Appendix 01 (PDF) [file pnas.2504774122.sapp.pdf]

PNAS

# Supporting Information for

Lysosomal Glucocerebrosidase is needed for ciliary Hedgehog signaling: A convergent pathway contributing to Parkinson's disease

Sreeja V. Nair, Ebsy Jaimon, Ayan Adhikari, Jonas Nikoloff, and Suzanne R. Pfeffer*

Corresponding Author: Suzanne Pfeffer, pfeffer@stanford.edu

**This PDF file includes:**

Figure S1. Immunoblot analysis of *Gba1* knockout in NIH-3T3 cells.

Table S1. Key Resources

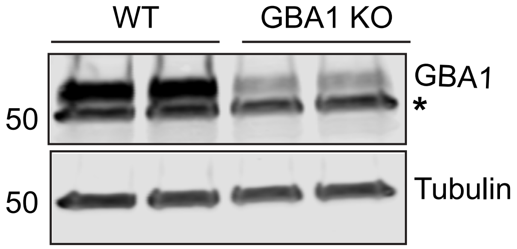


**Figure S1. Immunoblot analysis of *Gba1* knockout in NIH-3T3 cells.** Extracts from 40μg each of wild type (WT) and *Gba1* knockout NIH-3T3 fibroblasts were loaded in duplicate. Mass is shown at left in kDa and antigens are indicated at right; * indicates a non-specific band.

**Key Resource Table**

| Resource Type | Resource Name | Source | Identifiers | New/ Reuse | Additional information |
|---|---|---|---|---|---|
| | | | | | |
| Genetic reagent (Mus musculus) | C57BL/6N-Gba1tm1.1Mjff/J | The Jackson Laboratory | RRID:IMSR_ JAX:019 106 | Reuse | GBA1 D409V HOM |
| Antibody | anti-Arl13B (mouse monoclonal) | Neuromab | N295B/66 RRID:AB_2877361 | Reuse | IHC-(1:500), IF-(1:1000) |
| Antibody | anti-Choline Acetyltransferase (goat polyclonal) | Millipore | AB144P-1ML RRID:AB_2079751 | Reuse | (1:200) |
| Antibody | anti-ALDH1L1 (rabbit polyclonal) | Proteintech | 17390-1-AP RRID:AB_2878401 | Reuse | (1:200) |
| Antibody | anti-Adenylate cyclase III (rabbit polyclonal) | EnCOR | RPCA-ACIII RRID:AB_2572219 | Reuse | (1:10000) |
| Antibody | anti-Glucocerebrosidase (C-terminal, rabbit polyclonal) | Sigma-Aldrich | G4171 RRID:AB_1078958 | New | (1:1000) |
| Antibody | anti-alpha tubulin (mouse monoclonal) | Santa Cruz Biotechnology | sc-32293 RRID:AB_628412 | Reuse | (1:4000) |
| Antibody | H+L Donkey anti-Goat Alexa 488 | Life Technologies | A11055 RRID:AB_2534102 | Reuse | (1:2000) |
| Antibody | H+L Donkey anti-Mouse Alexa 568 | Life Technologies | A10037 RRID:AB_11180865 | Reuse | (1:2000) |
| Antibody | H+L Donkey anti-Mouse Alexa 488 | Life Technologies | A32766 RRID: AB_2762823 | Reuse | (1:2000) |
| Antibody | IRDye 800CW Donkey anti rabbit IgG | LI-COR | 926-32213 RRID:AB_621848 | Reuse | (1:10,000) |
| Antibody | IRDye 680RD Donkey anti mouse IgG | LI-COR | 926-68072 RRID:AB_10953628 | Reuse | (1:10,000) |
| Antibody | H+L Donkey anti-Rabbit Alexa 568 | Life Technologies | A10042 RRID:AB_2534017 | Reuse | (1:2000) |
| Commercial assay or kit | RNAscopeMultiplex Fluorescent Reagent Kit v2 | Advanced Cell Diagnostics | #323100 | Reuse | |
| Commercial assay or kit | RNAscope Probe-Mm- Gdnf | Advanced Cell Diagnostics | #421551 | Reuse | (1:10) |
| Commercial assay or kit | RNAscope Probe- Mm-Ptch1-C2 | Advanced Cell Diagnostics | #402811-C2 | Reuse | (1:5) |
| Commercial assay or kit | RNAscope Probe- Mm- Bdnf | Advanced Cell Diagnostics | #424821 | New | |
| Commercial assay or kit | OPAL 570 REAGENT PACK | Akoya Biosciences | FP1488001KT | Reuse | |
| Commercial assay or kit | OPAL 690 REAGENT PACK | Akoya Biosciences | FP1497001KT | Reuse | |
| Commercial assay or kit | High-Capacity cDNA Reverse Transcription Kit | Applied Biosystems | 4368814 | Reuse | |
| Software, Algorithm | FIJI | PMID:29187165 | RRID:SCR_002285 | Reuse | |
| Software, Algorithm | Prism | Prism 10 version 10.2.3 | RRID:SCR_002798 | Reuse | |

| | | | | | |
|---|---|---|---|---|---|
| Software, Algorithm | ZEN | Zeiss ZEN Microscopy Software | RRID:SCR_013672 www.zeiss.com/ microscopy/en/product s/softw are/zeiss-zen.html | Reuse | |
| Software, Algorithm | R | http://www.r-project.org/ | RRID:SCR_001905 | Reuse | |
| Software, Algorithm | Hisat2 v2.0.5 | | RRID:SCR_015530 | Reuse | |
| Software, Algorithm | featureCounts | v1.5.0-p3 PMID: 24227677 | RRID:SCR_012919 | Reuse | |
| Software, Algorithm | DESeq2 | Rpackage (1.20.0) doi.org/10.1186/s13059-014-0550-8 | RRID:SCR_015687 | Reuse | |
| Software, Algorithm | Gene Ontology | http://www.geneontology.org/ | RRID:SCR_002811 | Reuse | |
| Software, Algorithm | clusterProfiler | R package doi:10.1016/j.xinn.2021.100141. | RRID:SCR_016884 | Reuse | |
| Software, Algorithm | CellProfiler version 4.2.6 | PMID:29969450 | RRID:SCR_007358 | Reuse | |
| Software, Algorithm | Adobe Illustrator | Version 27.5 | RRID:SCR_010279 | Reuse | |
| Chemical compound, drug | Conduritol B epoxide (CBE) | Cayman Chemicals | 15216 | Reuse | 100μM, 7d |
| Chemical compound, drug | SAG | Cayman Chemicals | 11914 | Reuse | 25nM, 24h |
| Chemical compound, drug | U-18666A | Cayman Chemicals | 10009085 | Reuse | 1μM, 24h |
| Chemical compound, drug | Myriocin | Cayman Chemicals | 63150 | Reuse | 80 μM, 48h |
| Chemical compound, drug | Cholesterol | Sigma-Aldrich | C8667 | Reuse | |
| Chemical compound, drug | Methyl-β-cyclodextrin | Sigma-Aldrich | C4555 | Reuse | |
| Chemical compound, drug | SHH mouse Sonic hedgehog (residues 40–194, GenBank NP_033196) | Gift from Dr. Rajat Rohatgi, Stanford | DOI https://doi.org/10.1038/nsmb.1607 | Reuse | 10nM, 24h |
| Chemical compound, drug | PowerUp SYBR Green Master Mix | Applied Biosystems by Thermo Fisher Scientific | A25742 | Reuse | |
| Chemical compound, drug | Trizol reagent | Invitrogen | 15596026 | Reuse | |
| Chemical compound, drug | RNase OUT recombinant | Invitrogen | 100000840 | Reuse | |
| Chemical compound, drug | Paraformaldehyde Solution, EM grade | Electron Microscopy Sciences | 15710 | Reuse | |
| Chemical compound, drug | DMEM high glucose | Cytiva | SH30243.02 | Reuse | |
| Chemical compound, drug | Penicillin/Streptomycin | Cytiva | SV30010 | Reuse | |
| Chemical compound, drug | Fetal calf serum | Sigma | F0926 | Reuse | |
| Chemical compound, drug | Polybrene | Sigma | TR-1003-G | Reuse | |
| Chemical compound, drug | Polyethylenimine (PEI) | Polysciences | 23966 (1) | Reuse | |
| Cell line (mouse) | NIH-3T3-flpin | Thermo Fisher Scientific | R76107 (RRID:CVCL_U422) | Reuse | |
| Cell line (mouse) | MEF WT | MRC-PPU | from RRID: MMRRC_049312-UCD | Reuse | |

| | | | | | |
|---|---|---|---|---|---|
| Cell line (mouse) | MEF GBA1 D409V Hom | MRC-PPU | from RRID: MMRRC_049312-UCD | Reuse | |
| Cell line (human) | HEK293T | ATCC | RRID:CVCL_0063 | Reuse | |
| Cell line (human) | Fibroblast_healthy individual | Coriell Institute for Medical Research #GM08399 | RRID:CVCL_7482 | Reuse | |
| Cell line (human) | Fibroblast_healthy individual | Coriell Institute for Medical Research #GM04501 | RRID:CVCL_7409 | Reuse | |
| Cell line (human) | Fibroblast_Gaucher disease, type I | Coriell Institute for Medical Research #GM10915 | RRID:CVCL_0R39 | Reuse | GBA1 L444P |
| Cell line (human) | Fibroblast_Gaucher disease, type I | Coriell Institute for Medical Research #GM01607 | RRID:CVCL_7326 | Reuse | GBA1 N370S and V394L |
| Cell line (human) | Fibroblast_Gaucher disease, type II | Coriell Institute for Medical Research #GM00878 | RRID:CVCL_0R28 | Reuse | GBA1 L444P and A456P |
| Recombinant DNA reagent | PsPAX2 | Addgene | RRID:Addgene_12260 | Reuse | |
| Recombinant DNA reagent | VSV-G | Addgene | RRID:Addgene_12259 | Reuse | |
| Recombinant DNA reagent | mApple-SSTR3-N-17 | Addgene | RRID:Addgene_54949 | Reuse | |
| Raw data for figures_this paper | | Zenodo | https://doi.org/10.5281/zenodo.12753029, https://doi.org/10.5281/zenodo.15801264 | New | |
| Protocol | Small scale Lentivirus Production and Infection | Protocols.io Aligning Science Across Parkinson's | https://dx.doi.org/10.17504/protocols.io.bp2l61z2zvqe/v1 | Reuse | |
| Protocol | Detection of accessible cholesterol in primary cilia using purified His- mNeon-ALOD4 inNIH- 3T3 Fibroblasts | Protocols.io Aligning Science Across Parkinson's | https://dx.doi.org/10.17504/protocols.io.rm7vzx2qxgx1/v1 | Reuse | |
| Protocol | An ImageJ macro for the quantification of ciliary accessible cholesterol V.2 | Protocols.io Aligning Science Across Parkinson's | https://dx.doi.org/10.17504/protocols.io.q26g71d88gwz/v2 | Reuse | |
| Protocol | Immunostaining and quantification of intracellular accessible cholesterol using His- mNeon-ALOD4 in Human Fibroblasts | Protocols.io Aligning Science Across Parkinson's | https://dx.doi.org/10.17504/protocols.io.rm7vzj5prlx1/v1 | Reuse | |
| Protocol | Immunofluorescent Staining of phosphoRab10 in cultured cells | Protocols.io Aligning Science Across Parkinson's | https://dx.doi.org/10.17504/protocols.io.ewov1nmzkgr2/v1 | Reuse | |
| Protocol | A method for RNA extraction, cDNA synthesis and and quantitative PCR from NIH-3T3 fibroblasts | Protocols.io Aligning Science Across Parkinson's | https://dx.doi.org/10.17504/protocols.io.rm7vzjo84lx1/v1 | Reuse | |
| Protocol | PCR and analysis | Protocols.io Aligning Science | https://dx.doi.org/10.17504/protocols.io.kxygxypwdl8j/v1 | Reuse | |

| | | | | | |
|---|---|---|---|---|---|
| | | Across Parkinson's | | | |
| Protocol | Creation of pooled CRISPR KO cell lines using Synthego sgRNA | Protocols.io Aligning Science Across Parkinson's | https://dx.doi.org/10.17504/protocols.io.bp2l6dqodvqe/v1 | Reuse | |
| Protocol | Immunohistochemistry in Rodent Brain | Protocols.io Aligning Science Across Parkinson's | dx.doi.org/10.17504/protocols.io.bnwimfce | Reuse | |
| Protocol | Fluorescence in situ hybridization (FISH) | Bioprotocol | https://bio-protocol.org/exchange/protocoldetail?id=1423&type=3 | Reuse | |
